# Cryptic temporal changes in stock composition explain the decline of a flounder (*Platichthys* spp.) assemblage

**DOI:** 10.1111/eva.12738

**Published:** 2019-01-21

**Authors:** Paolo Momigliano, Henri Jokinen, Federico Calboli, Eero Aro, Juha Merilä

**Affiliations:** ^1^ Ecological Genetics Research Unit, Research Program in Organismal and Evolutionary Biology University of Helsinki Helsinki Finland; ^2^ Tvärminne Zoological Station University of Helsinki Hanko Finland; ^3^ Department of Biology University of Leuven Leuven Belgium; ^4^ Finnish Game and Fisheries Research Institute Helsinki Finland; ^5^ Present address: Puolipäivänkatu 4 A 6, FI‐00160 Helsinki Finland

**Keywords:** Baltic Sea, climate change, eutrophication, fishery, flounder, mixed-stock, otolith, unobserved diversity

## Abstract

Unobserved diversity, such as undetected genetic structure or the presence of cryptic species, is of concern for the conservation and management of global biodiversity in the face of threatening anthropogenic processes. For instance, unobserved diversity can lead to overestimation of maximum sustainable yields and therefore to overharvesting of the more vulnerable stock components within unrecognized mixed‐stock fisheries. We used DNA from archival (otolith) samples to reconstruct the temporal (1976–2011) genetic makeup of two mixed‐stock flounder fisheries in the Åland Sea (AS) and the Gulf of Finland (GoF). Both fisheries have hitherto been managed as a single stock of European flounders (*Platichthys flesus*), but were recently revealed to target two closely related species: the pelagic‐spawning *P. flesus* and the newly described, demersal‐spawning *P. solemdali*. While the AS and GoF fisheries were assumed to consist exclusively of *P. solemdali*, *P. flesus* dominated the GoF flounder assemblage (87% of total) in 1983, had disappeared (0%) by 1993, and remained in low proportions (10%–11%) thereafter. In the AS, *P. solemdali* dominated throughout the sampling period (>70%), and *P. flesus* remained in very low proportions after 1983. The disappearance of *P. flesus* from the GoF coincides in time with a dramatic (~60%) decline in commercial landings and worsening environmental conditions in *P. flesus’* northernmost spawning ground, the Eastern Gotland Basin, in the preceding 4–6 years. These results are compatible with the hypothesis that *P. flesus* in the GoF is a sink population relying on larval subsidies from southern spawning grounds and the cause of their disappearance is a cessation of larval supply. Our results highlight the importance of uncovering unobserved genetic diversity and studying spatiotemporal changes in the relative contribution of different stock components, as well as the underlying environmental causes, to manage marine resources in the age of rapid anthropogenic change.

## 1 INTRODUCTION

Unobserved genetic diversity, whether in the form of within‐species genetic structuring or of the presence of cryptic species (morphologically indistinguishable biological groups incapable of interbreeding), is of major importance for the conservation and management of global biodiversity in the age of rapid environmental changes (Bickford et al., 2007; Dirzo et al., 2014). Biodiversity assessment at the level of morphospecies (species that can be distinguished by quantifiable morphological characteristics) is likely to greatly underestimate species diversity across taxonomic groups and biogeographic regions (Pfenninger & Schwenk, 2007), and thus hamper the estimate of biodiversity loss due to environmental changes (Bálint et al., 2011). Unveiling hidden genetic structure resulting from local adaptation (Benestan et al., 2015; Bradbury et al., 2013; Lamichhaney et al., 2012; Momigliano et al., 2017a; Prunier, Laroche, & Bousquet, 2011) and cryptic speciation (Bradbury et al., 2014, 2010; Fennessy et al., 2016; Herbert et al., 2004; Momigliano, et al., 2017b) is important in the delineation of conservation and management units for exploited or threatened species (Funk et al., 2012), as well as for the identification of suitable protected areas that maximize biodiversity representativeness and complementarity (Cook, Page, & Hughes, 2008). Cryptic species complexes that consist of rare or threatened taxa are especially vulnerable, as each taxon can be even rarer or more threatened than the one nominal unit being considered and may respond differently to environmental change (Schönrogge et al., 2002). Unveiling cryptic species and populations also enables more accurate estimates of taxon‐specific biological traits (e.g., Feckler et al., 2014) that determine resilience to environmental change and exploitation (Hilborn, Quinn, Schindler, & Rogers, 2003). The presence of cryptic taxa poses serious challenges to understanding the causes and effects of population change, since it can confound our understanding of past and present demographic changes, source–sink dynamics, population connectivity, and trophic interactions.

Unobserved genetic diversity has especially important implications for the management of exploited marine resources. Fishery management has been based on the assessment of stocks, usually defined as genetically homogeneous and demographically independent populations which were often unrealistically assumed to have clear and temporally stable geographic boundaries (Begg, Friedland, & Pearce, 1999). The past three decades of fishery research demonstrate that this is seldom the case: single‐species fisheries may exploit multiple, demographically independent but morphologically indistinguishable stocks that at times co‐occur in the same geographic location (Bonanomi et al., 2015; Campana, Chouinard, Hanson, & Fréchet, 1999; Jónsdóttir, Marteinsdottir, & Campana, 2007; Lindegren, Waldo, Nilsson, Svedäng, & Persson, 2013; Schuchert, Arkhipkin, & Koenig, 2010). A failure to recognize this unobserved diversity can lead to overestimated maximum sustainable yields, and therefore to overharvest of the more vulnerable stock components (Hutchinson, 2008; Sterner, 2007). Examples of mixed‐stock fisheries include the cod fisheries in the Gulf of St. Lawrence (Ruzzante, Taggart, Lang, & Cook, 2000) and the Kattegat (Lindegren et al., 2013), which were traditionally considered as single‐stock fisheries but turned out to be mixtures of stocks that aggregate during feeding but segregate during spawning.

Differences between stock components may be behavioral (e.g., different spawning ecotypes) and/or genetic (locally adapted populations) instead of clearly morphological and are therefore likely to be overlooked by fishery managers and fishermen (Reiss, Hoarau, Dickey‐Collas, & Wolff, 2009). Furthermore, cryptic species or unrecognized populations of the same species within a mixed‐stock fishery may respond differently to fishing pressure and to natural and anthropogenic environmental change, leading to undetected spatiotemporal shifts in their relative contribution to local stocks (Bonanomi et al., 2015). Such processes may lead to dramatic fishery collapse, as demonstrated in a recent study on the West Greenland cod fishery (Bonanomi et al., 2015). Using diagnostic SNPs genotyped from archived samples, Bonanomi et al. (2015) reconstructed the spatiotemporal relative contribution of two biologically distinct (but morphologically indistinguishable) major stock components: the local and cold‐adapted West Greenland offshore cod population and the Iceland offshore cod population, the contribution of the latter being correlated with higher sea surface temperature (SST). The authors revealed that the collapse of the West Greenland cod fishery in the 1970s was the result of the gradual disappearance of the local West Greenland offshore population (due to overfishing), and the subsequent decline of Iceland offshore cod due to a period of unfavorable (colder) SST.

The fact that separate stock components may respond very differently to environmental change and fishing pressure is of concern in the age of rapid climate change (Hoegh‐Guldberg & Bruno, 2010), coastal eutrophication (Smith, Tilman, & Nekola, 1999), and overfishing (Pauly, Christensen, Dalsgaard, Froese, & Torres, 1998). Spatiotemporal tracking of the contribution of different genetic populations to mixed‐stock fisheries (Bonanomi et al., 2015; Dahle, Johansen, Westgaard, Aglen, & Glover, 2018; Ruzzante et al., 2000) may therefore play a pivotal role in future adaptive management aimed at avoiding further fishery collapse that may be only partially dependent on local fishing pressure and may be affected by environmental changes occurring in spawning areas hundreds of kilometers away from the fishing grounds.

**Figure 1:**
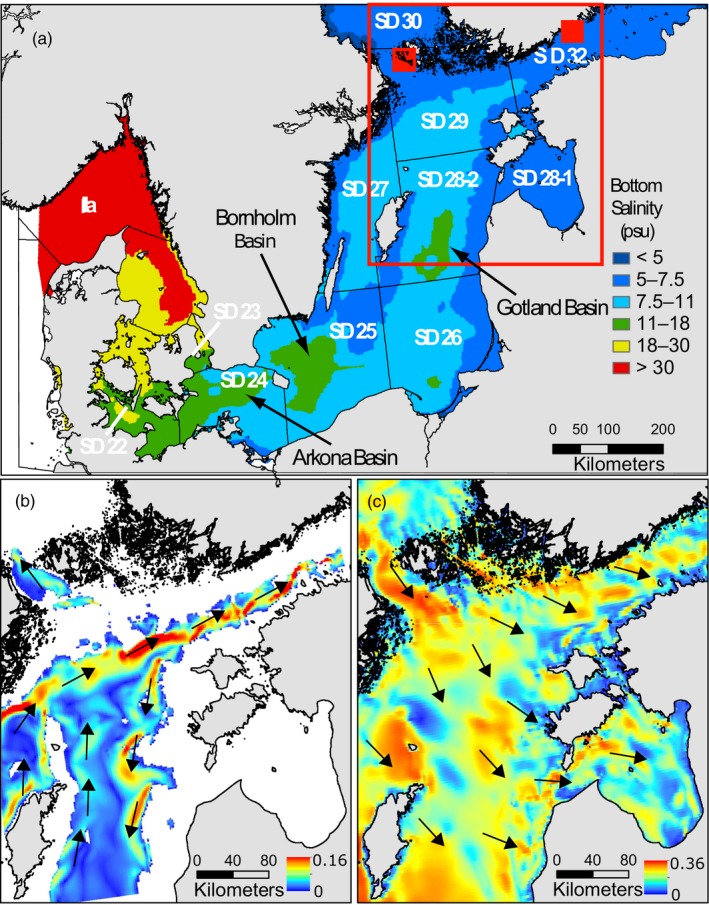
(a) Map of the Baltic Sea showing International Council for the Exploration of the Sea (ICES) Subdivisions (SD) 23–32 and modeled mean bottom salinity from Bendtsen, Söderkvist, Dahl, Hansen, and Reker (2007). Areas colored in red, yellow, and green are theoretically suitable spawning habitats for *P. flesus*. The three major spawning grounds of *P. flesus* in the Baltic Sea are indicated by black arrows. Red filled squares represent sampling areas in the AS (SD 29) and the GoF (SD 32). The large red rectangle identifies the main study area, including the EGB, the AS, and the GoF. (b and c) show absolute water velocity (m/s) in spring in the main study area at depths of 70 m (b) and 10 m (c). The arrows represent current direction. Data for b and c represent monthly means for May 2017 and were retrieved from the European Union's Copernicus Marine Monitoring Service. Additional data for other periods and depths are shown in the Supporting information Figures S2-S5

Flounders (*Platichthys* spp.) in the Baltic Sea show two distinct reproductive strategies: offshore spawning of pelagic eggs and coastal spawning of demersal eggs (Nissling, Westin, & Hjerne, 2002; Solemdal, 1967). Pelagic spawning occurs exclusively in deep offshore basins of the southern and central Baltic Sea (*viz*. the Arkona Basin, the Bornholm Basin, and the Eastern Gotland Basin) where salinity is sufficiently high (>11 psu, see Figure 1a) for eggs to achieve neutral buoyancy (Nissling et al., 2002). Flounders with demersal eggs can spawn successfully in salinities as low as 6 psu (Nissling et al., 2002), in conditions routinely encountered in coastal waters of the Northern Baltic Proper (NBP) and the Gulf of Finland (GoF).

In a recent study, Momigliano et al. (2017b) demonstrated that Baltic Sea flounders with pelagic and demersal eggs are a pair of closely related species arising from two independent colonization events of the Baltic Sea from the same ancestral population following the end of the last glaciation. The Baltic Sea flounders with demersal eggs have since been officially described as a new species: the Baltic flounder *Platichthys solemdali* (Momigliano, Denys, Jokinen, & Merilä, 2018). Based on studies from the past decade, European (*P. flesus*) and Baltic (*P. solemdali*) flounders are considered parapatric, with both species co‐occurring in some areas of the central Baltic Sea. For example, the species meet around Gotland, which is assumed to be the northern limit of *P. flesus*’ distribution (Florin & Höglund, 2008; Hinrichsen et al., 2017; Nissling et al., 2002), and at the southwestern entrance of the Gulf of Riga, where *P. flesus* and *P. solemdali* co‐occur in similar proportions (Momigliano et al., 2017b).

The two flounder species are fished throughout the Baltic Sea, where they are caught both as by‐catch in the cod fishery and directly targeted using gillnets. In 2014, the International Council for the Exploration of the Sea (ICES) convened the Benchmark Workshop on Baltic Flatfish Stocks (WKBALFLAT, ICES, 2014), which separated the Baltic flounder fishery into four assessment and management units: ICES subdivisions (SDs) 22–23, SDs 24–25, SDs 26 and 28, and SDs 27 and 29–32 (see Figure 1a for a map of ICES SDs). The first three are assumed to be dominated by *P. flesus *(but it is recognized that both species co‐occur in SD 28), while the flounder fishery in the Northern Baltic Proper and the Gulf of Finland (SD 27 and SDs 29–32) is considered a single stock of *P. solemdali *with no significant contribution *of P. flesus. *Within SD 27 and 29–32, most of the fishing effort is concentrated in two areas: the Åland Sea (AS, the northwestern part of SD 29 between the Åland Archipelago and the Swedish coast, Figure 1a) and the Gulf of Finland (the entire SD32, Figure 1a), where flounders are targeted mainly by Estonian and Finnish fishing fleets (ICES, 2014). In the past ten years, catches in SD 27 and 29–32 averaged at approximately 200 tonnes per year (t/y), but in the first few years for which yearly estimates are available (1980–1984), catch estimates were above 1,500 t/y and started to decline in the second half of the 1980s. The most dramatic decline in landings took place in the GoF: In the early 1980s, more than 1,000 t/y were landed, whereas in the last ten years, landings averaged below 100 t/y (ICES, 2017). While there are some doubts on how reliable catch estimates in the 1980s were, particularly from the USSR, catch per unit effort (CPUE) data from fish surveys on the Finnish coast confirm a decrease of over 60% from 1975 to 1995, and over 90% decrease from 1975 to 2012 (Jokinen et al., 2015), suggesting that the temporal decline in landings was not entirely a reflection of inaccurate reporting or lower fishing effort.

Momigliano et al. (2018, 2017b) discovered that *P. flesus* can, in fact, be found at low density throughout the NBP (including the AS) and the GoF, and that they do not interbreed with *P. solemdali* (both studies found no evidence of hybridization in >300 individuals sampled throughout the Baltic Sea). The co‐occurrence of both species has been likely overlooked because *P. flesus* and *P. solemdali* can only be distinguished based on genetic data, gamete morphology, and physiological differences that cannot be routinely assessed by fishers and managers (Momigliano et al., 2018). One explanation for the occurrence of *P. flesus *in the AS and GoF, where there are no suitable spawning grounds (Figure 1a), is that these locations are “sinks” (Pulliam, 1988; Underwood & Fairweather, 1989), sourced through either larval subsidies or adult spillover from spawning grounds in the central Baltic Sea—as is the case for cods in the Gulfs of Finland and Riga (Aro, 1989; Casini et al., 2012). This discovery raises the question of what the historical contribution of immigration from southern spawning grounds to local flounder stocks in the AS and GoF was, and whether changes in immigration of *P. flesus* from more productive areas in the central Baltic Sea may be related to local declines in the GoF (Jokinen et al., 2015; Jokinen, Wennhage, Ollus, Aro, & Norkko, 2016).

In the northernmost spawning ground for *P. flesus*, the Eastern Gotland Basin (EGB), the reproductive volume (RV, i.e., the volume of water where [O_2_] and salinity are suitable for spawning) fluctuates through time based on changes in salinity and oxygen content following major saltwater influxes and stagnation periods (Hinrichsen et al., 2017; Nissling et al., 2002; Ustups, Müller‐Karulis, Bergstrom, Makarchouk, & Sics, 2013). At the same time, eutrophication and climate change are causing a rapid increase in hypoxia and anoxia in bottom waters of the Baltic Sea (Carstensen, Andersen, Gustafsson, & Conley, 2014). Consequently, the suitable spawning habitat of *P. flesus*, which has always been geographically limited to higher‐salinity deep‐water spawning grounds (Nissling et al., 2002, see also Figure 1a), has contracted in recent decades, resulting in fluctuations in both stock size (Orio et al., 2017; Ustups et al., 2013) and larval survival (Hinrichsen et al., 2017), particularly in the EGB. This could have reduced larval subsidies and adult spillover to the Gulf of Finland, thus providing a plausible explanation for the declining trend seen in this area. However, changes in local environmental conditions (i.e., temperature, salinity) affecting reproduction of *P. solemdali* in the GoF and a decline in habitat quality of local nursery areas have also been suggested as potential causes of recent local declines in the Gulf of Finland (Jokinen et al., 2015, 2016; Ojaveer & Kalejs, 2005).

Here, we used genetic data from archival (otolith) samples to reconstruct the temporal genetic makeup of a mixed flounder stock in the Åland Sea (AS) and the Gulf of Finland (GoF), which has hitherto been managed as a single stock of the European flounders (*P. flesus*) (ICES, 2017), but was recently revealed to consist of two closely related species (Momigliano et al., 2018, 2017b). Our main aim was to test for possible temporal changes in stock composition in coastal areas of the Northern Baltic Sea. In particular, we explored the hypothesis that *P. flesus* from the EGB are a source population seeding the GoF, and that past fluctuations in stock size were influenced by environmental changes in geographically distant source populations. Specifically, we wanted to know how the relative proportions of *P. flesus* and *P. solemdali* have changed over time in the study region, and how these changes relate to RV in the EGB. To this end, we reconstructed the relative proportion of *P. flesus* and *P. solemdali* over the past four decades in two locations, one likely to receive larval subsidies from the EGB (GoF), and another that is less likely to receive larval subsidies (AS) given the prevailing water current patterns in the Baltic (Maslowski & Walczowski, 2002). We hypothesized that if larval subsidies of *P. flesus* from the central Baltic are an important factor in the demographics of flounders in the GoF: (a) the proportion of *P. flesus* in the GoF, but not in the AS, should be correlated with environmental conditions favorable to pelagic spawning in the EGB, (b) there should be a temporal decrease in the proportion of *P. flesus* in the GoF reflecting worsening environmental conditions in the central Baltic Sea, and (c) this temporal trend should coincide in time with the reported stock decline in GoF.

## 2 MATERIALS AND METHODS

### 2.1 The otolith collection

To determine the relative contribution of *P. solemdali* and *P. flesus *to local populations, we used selected samples from a collection of more than 29,000 flounder otoliths, which have been collected as part of sampling by the Finnish Game and Fisheries Research Institute (now the Finnish Natural Resources Institute, Luonnonvarakeskus, LUKE) annually from 1975 to 2011.

In 1975–1998, primary data were collected mainly from flounder commercial gillnet catches (mesh size 60–80 mm) and gillnet flounder by‐catches (mesh size 45–55 mm) targeted for other species (e.g., pike‐perch, perch, whitefish, and bream). Sampling was conducted as a random sampling design during the main fishing seasons (mainly year quarters 3 and 4) by sampling whole daily flounder catch/fisherman (catches varied between 10 and 50 kg per fisherman). In both areas, length and age compositions of catches and by‐catches were recorded for stock assessment. Both sagittal otoliths were sampled for age determination. This sampling scheme was connected to ICES flounder stock assessment and monitoring of the state of the flounder stocks in the Baltic.

In 1999–2011, the sampling scheme was changed by an internationally coordinated sampling program in order to standardize sampling between countries and areas. As before, the whole daily flounder catch/fisherman was recorded, and details of gillnets and their properties were recorded. Sample size varied between 10 and 50 kg per fisherman. A normal length‐based stratified sampling strategy was used for otoliths. The whole catch was weighted and specimens measured according to 1 cm classes. For age determination, the aim was to collect 10 otoliths/each cm length class per year quarter (quarters 3 and 4).

Whole sagittal otoliths were used for age determination. All otoliths were read only by one person 1975–2004 for subdivisions 29–30 (see Figure 1 for a map of ICES subdivisions in the Baltic Sea) and throughout the whole sampling period for subdivision 32, to minimize age reading error. Having both sampling date and age estimate from each sample, the birth year of each individual fish could be determined. We obtained DNA from 480 individuals, representing multiple time points and cohorts within two sampling areas (see below). The rationale for selecting sampling areas and for sub‐sampling individuals are outlined in the two following sections.

### 2.2 Sampling areas

Two areas, representing the Åland Sea (the western Åland Archipelago; Finnish National Square 49) and the Gulf of Finland (the Helsinki area; Finnish National Squares 54/53), were selected for the study (henceforth referred to as “AS” and “GoF,” respectively; Figure 1). The two sampling locations were selected for two main reasons. First, in both areas we had access to a large number of samples obtained at multiple time points, and we were therefore able to obtain reasonable sample sizes (≥48 individuals) at regular intervals for the past decades (see next paragraph for details). Second, and most importantly, based on bathymetry and water circulation patterns (see Figure 1b,c and Supporting information Figures S2–S5) we predict that larval subsidies from the EGB are likely to be transported by currents to the GoF, but not to the AS. The low salinity precludes the possibility of pelagic spawning in both areas (Figure 1a), and hence, *P. flesus* occurring in the AS and the GoF are likely to be the result of either larval subsidies or adult spillover from the EGB. *P. flesus* larvae are spawned in deep waters of the EGB, and they can maintain neutral buoyancy only below the 10 psu isohaline. Initial dispersal is therefore expected to happen along deep‐water currents. Prevailing deep‐water currents in spring flow northward in the Easter Gotland Basin (Yi et al., 2013). South of the Åland archipelago, the depth of the Northern Baltic Sea decreases to less than 60 m, and the deep‐water current flowing northward from the EGB is deflected eastward toward the GoF (Yi et al., 2013; Figure 1b). While we do not have access to circulation data for past four decades, data from the past two decades (accessed through E.U. Copernicus Marine Service Information, http://marine.copernicus.eu/) suggest this pattern is consistent through time (see examples in Figure 1b and Supporting information Figures S2–S5). Therefore, deep‐water currents could transport pelagic propagules from southern spawning grounds into the GoF, but not to the AS. In the western Åland archipelago, there is also a prevailing and strong north to south flow of surface freshwater from the Bothnian Bay in spring (Maslowski & Walczowski, 2002; Yi et al., 2013, see also Figure 1c and Supporting information Figures S2–S5), which would present an additional barrier to the northward dispersal of flounder eggs and larvae.

### 2.3 Time points and cohorts

For both areas, four time points were sampled (Table 1). These time points were chosen to ensure sampling occurred as much as possible at regular intervals within the past four decades, (a) including periods before and after the decline in landings and CPUE in the GoF in the 1980s, and (b) so that the samples’ birth years included times in which the EGB RV was very high (e.g., the late 1970s), very low (late 1980s) and somewhere in between (late 1990s and 2000s, see Ustups et al., 2013). In the GoF area, we sampled the Finnish National Square 54 for every year except 1992, when the adjacent square (Finnish National Square 53) was used instead to secure the required number of samples.

**Table 1:** Number of genotyped individuals from each Finnish National Square in different sampling years. Cohort refers to the year of birth of sampled fish as determined by otolith analyses

Finnish National Square	Year of sampling	Cohort	*N* genotyped
49	1976	1970	36
49	1976	1971	30
49	1983	1977	24
49	1983	1978	22
49	1992	1986	22
49	1992	1987	22
49	2002	1997	24
49	2003	1997	6
49	2002	1998	24
49	2003	1998	9
54	1983	1973	4
54	1983	1974	17
54	1983	1975	25
54	1983	1977	11
54	1983	1978	14
54	1983	1979	22
53	1992	1986	16
53	1992	1987	19
54	2003	1997	23
54	2003	1998	24
54	2011	2006	25

We extracted DNA from a total of 480 otolith samples, aiming at a minimum number of 48 samples per area for each sampling time point. From each study area, we sampled 24 individuals from two distinct cohorts (either 4‐ and 5‐ or 5‐ and 6‐year‐old fish), with the exception of year 1983 in square 54 from which we sampled five cohorts. The 24 individual otoliths analyzed for each cohort and sampling location combinations were chosen by randomly sampling individuals for each cohort and sampling location combination available in LUKE's otolith collection. This way we obtained data for all in all 9 and 11 birth years for the AS and the GoF sampling areas, covering time periods of 1970–1998 and 1973–2007, respectively. DNA was extracted using a modified salting out procedure, optimized for otolith samples (see protocol in the Supporting information).

### 2.4 Genotyping

Five SNPs (SNPs 3556_15, 1822_9, 3599_4, 4474_34, and 886_19) that had been previously identified as under divergent selection in the two species (Momigliano et al., 2017b) and able to unambiguously assign unknown individuals to the correct species (Momigliano et al., 2018) were genotyped using a SNaPshot™ Multiplex Kit (Thermo Fisher). All loci were amplified in a single, multiplexed PCR using the primers developed by Momigliano et al. (2018) targeting the regions flanking the outlier loci (GenBank Accession numbers: KY933571–KY933582) sequenced by Momigliano et al. (2017b). The primers target very short DNA fragments (~100 bp in length), enabling amplification from highly degraded DNA recovered from otoliths. SNPs were genotyped using the SNaPshot™ probes designed by Momigliano et al. (2018), following manufacturer's instructions.

### 2.5 Assignment tests

We employed a Bayesian approach where the probability of a fish to be assigned to one species is calculated as a function of its genotype and prior belief that the sample belongs to a given species; this probability is then updated as more genetic tests are performed in succession. The very same Bayesian approach has been used by Toli, Calboli, Shikano, and Merilä (2016) for sex identification after multiple consecutive genetic tests, and the full conceptual framework and the mathematical formulation of this approach are detailed in that paper. Because every sample can be drawn from two possible species, calculating the probability that a sample has been drawn from one immediately provides also the probability that the sample has been drawn from the other, because *p* *(P. solemdali*) = 1 – *p* (*P. flesus*).

Briefly, we determined the allelic and genotypic frequencies at five loci for *P. flesus *and *P. solemdali* based on 235 samples (128 *P. solemdali* and 107 *P. flesus*) whose species identities had been previously determined with certainty (see Momigliano et al., 2017b; data available from Momigliano et al., 2017c); this information provided us with the specificity and sensitivity of each genotype for attributing each sample to one (or the other) species using the approach described by Toli et al. (2016). We chose a conservative (uninformative) prior of *p* = 0.5, and we used the genotype of the sample at a first locus and its relative frequency in the two species to calculate a posterior probability that the sample was drawn from *P. solemdali*. This posterior probability was then used as the prior for the next test, based on the sample's genotype at the next marker and its relative frequency in the two species. The result of this calculation was then an updated posterior probability that the sample belongs to *P. solemdali*. This iterative process was carried for all subsequent genetic tests, allowing us to update and refine the posterior probability. Samples whose final posterior probability of being *P. solemdali* was 1 (or close to one) can be considered as the demersal‐spawning species with a high degree of confidence, and samples whose final posterior probability of being *P. solemdali* was 0 (or close to 0) could be confidently identified as *P. flesus*.

### 2.6 Environmental drivers

To test whether environmental conditions in the EGB could explain the observed temporal change in the proportion of the two species in GoF and AS, we looked for a relationship between the relative abundance of *P. flesus* in the study areas and the RV in the EGB for the year in which the fish of each cohort were born (“birth year”). The RV describes the spawning and early life conditions of pelagic flounders, and is defined as the volume of water where salinity and oxygen conditions are suitable for spawning and for the survival of eggs and larvae (i.e., 10.7–12 psu and [O2] of >1 ml/L). Estimates of RV calculated at bimonthly intervals for the period 1970–2005 were obtained from Figure 2 in Ustups et al. (2013), for each year in which sampled fish were born. The time of the year expected to be most important in determining flounders' spawning success and propagule survival is May–June, the period during which spawning and early development occur (Bagge, 1981; Hinrichsen et al., 2017; Nissling, Nyberg, & Petereit, 2017). However, environmental conditions in earlier months, when aggregation at spawning sites may start, could also be important: for example, hypoxic conditions could disturb aggregations of adult fish at spawning sites. We therefore looked for a linear relationship between the percentage of *P. flesus* in the GoF and AS and the RV at and immediately after the time of spawning (May–June of the year of birth), as well as in the preceding (March–April) and following months (July–August), using major axis regression (reflective of the fact that both dependent and independent variables are measured with error). The same data were also analyzed using a logistic regression to determine whether there is a “threshold” in RV after which most of the individuals are likely to be *P. flesus*. In this analysis, each individual was treated as an observation (i.e., it can be either *P. solemdali* or *P. flesus*): the probability of an individual being *P. flesus* was the dependent variable, while RV in the EGB was the explanatory variable. Analyses were performed separately for the two sampling locations. P‐values were adjusted for multiple comparisons using the Holm–Bonferroni correction. All statistical analyses were performed using the R computing environment (R Core Team, 2017).

**Figure 2:**
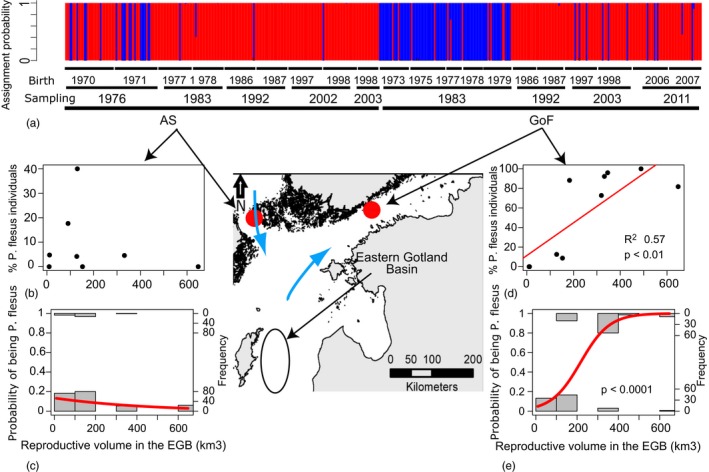
(a) Bayesian assignment test based on five outlier SNPs. Each bar represents an individual. *Y*‐axis represents the assignment probability to the demersal‐spawning *P. solemdali* (red) and pelagic‐spawning *P. flesus* (blue). On the *x*‐axes are given the sampling location, sampling year, and the birth year (sampling year minus fish age estimated from otolith). The map shows sampling locations (AS and GoF) and the potential “source” population (EGB), arrows show dominant current patters (Maslowski & Walczowski, 2002). (b–e) Relationship between reproductive volume in the EGB (Ustups et al., 2013) and proportion of *P. flesus*. There is no linear (b) or logistic (c) relationship in the AS, while in the GoF (d), reproductive volume explained 57% of the variation in the proportion of *P. flesus*. (e) the same data analyzed using a logistic regression to determine whether there is a “threshold” in reproductive volume after which most of the individuals are likely to be the pelagic‐spawning species

## 3 RESULTS

A total of 444 individuals were successfully genotyped, out of which 433 were identified to species level with more than 99% probability and 439 with more than 95% probability (Figure 2). If a 95% posterior probability is chosen as a threshold for assignment, only 1% of the samples remain of uncertain identity. In both the GoF and the AS, the proportion of *P. flesus* was lower in contemporary samples than at the start of the time series (Figure 2a). In the GoF, *P. flesus* dominated the flounder assemblage (87% of total) in 1983, had disappeared entirely in 1993, and showed low proportions (10%–11%) in 2003 and 2011 (Figure 2a). In the AS, *P. solemdali* was dominant throughout the sampling period (>70% of total; Figure 2). In 1976, *P. flesus* contributed a larger proportion (30% of total) to the local assemblage, but this was largely due to a single cohort (40% of fish born in 1971 were *P. flesus* vs. only 18% of fish born in 1970), and already in 1983, the proportion of *P. flesus* had dramatically declined and showed similarly low occurrence (2% of total) in all remaining sampling time points (Figure 2a).

**Figure 3:**
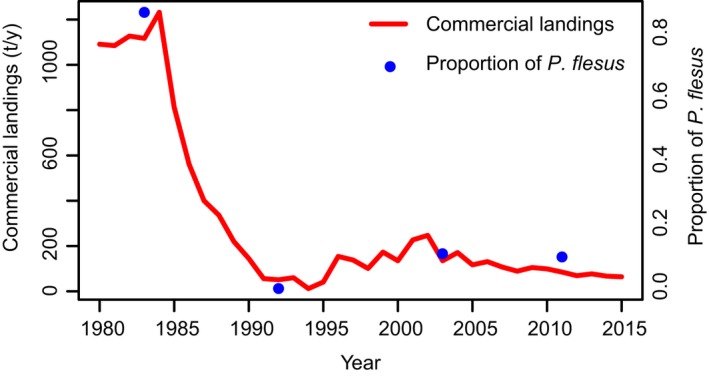
The proportion of *P. flesus* in the GoF (SD 32) and the total commercial landing (ICES, 2017) as a function of time

There was a positive relationship between the proportion of *P. flesus* in the GoF and the RV in the EGB in May–June (Holm–Bonferroni adjusted *p* = 0.042, adjusted *R* ^2^ = 0.57, Figure 2d), but not with the RV in March–April and July–August (*p* > 0.05, Supporting information Figure S1). The relationship between the RV in May–June and the proportion of *P. flesus* in the GoF was not perfectly linear, and logistic regression analyses (Holm–Bonferroni adjusted *p* = 8.7 × 10^−12^, *r* ^2^ = 0.575) suggest that when the RV in the EGB increases over 300 km^3^, there is an abrupt increase in the probability of sampling *P. flesus* individuals (Figure 2e). As expected, there was no relationship (*p* > 0.05) between EGB reproductive volume and the proportion of *P. flesus* in the AS (Figure 2b,c, Supporting information Figure S1). When comparing the flounder landings from the GoF (ICES, 2017) to the proportion of *P. flesus* in the GoF samples, there is an almost perfect concordance between the two (Figure 3), so that landings were higher at times when the proportion of *P. flesus* in the GoF was higher, and the abrupt decline in landings closely matches in time the shift from a *P. flesus*‐dominated to *P. solemdali*‐dominated flounder assemblage.

## 4 DISCUSSION

It has long been assumed that only demersal‐spawning flounders (*P. solemdali*) occur in the GoF and that the two species only co‐exist in the Central Baltic Sea (ICES, 2017). Our results demonstrate that, as was suggested by Momigliano et al. (2017b; 2018), this is not the case. Both species co‐occur in the AS and the GoF, and at different times, either species can dominate local assemblages. There was a major temporal shift in the relative proportion of *P. flesus* and *P. solemdali* in the GoF that was correlated with changes in environmental conditions in the EGB (Nissling et al., 2002; Ustups et al., 2013). In the GoF, *P. flesus* made up the overwhelming majority of the flounders in all the cohorts from 1973 to 1979, and since the late 1980s, they consistently contributed to less than 10% of the individuals (see also: Florin & Höglund, 2008, Momigliano et al., 2017b, Momigliano et al., 2018). These results reveal that the flounders of the GoF not only do not represent a single entity and fishery stock, but are indeed a mixture of two distinct species, each of which can dominate local assemblages at different points in time. These results highlight the fact that defining fishery stocks and management and conservation units based exclusively on the contemporary distribution of ecological and genetic diversity may lead to erroneous assessment, as this may represent a snapshot in time of a complex, dynamic picture.

The shift from *P. flesus‐* to *P. solemdali*‐dominated assemblages coincides with the timing of the decline in GoF landings (ICES, 2017), as well as with a sharp decline in flounder CPUE in fishery‐independent Finnish coastal fish surveys between 1975 and 1995 (Jokinen et al., 2015). Since salinity in the GoF is too low for *P. flesus* reproduction even under the most favorable conditions (Nissling et al., 2002), these findings suggest that either larval supply or juvenile and adult spillover from southern populations may have been important factors in shaping flounder abundance in the GoF. The fact that a similar temporal shift was not observed in the AS, which is not reached by northward deep‐water currents from the EGB and where the prevailing surface currents flow southward and therefore are unlikely to carry larval subsidies from the south, brings further support to the hypothesis that changes in larval supply, rather than reduced adult spillover, are the main underlying cause of the shift from *P. flesus* to *P. solemdali* dominance in the GoF. The observed change in the relative contribution of the two flounder species could also be explained by an increase in the local population of *P. solemdali*. However, this is unlikely, since the shift in dominance from *P. flesus* to *P. solemdali* has happened against the backdrop of an overall decline in flounder landings and CPUE in the GoF (ICES, 2017; Jokinen et al., 2015).

A previous study demonstrated that larval abundance of flounders in the EGB is correlated with both spawning stock biomass (SSB) and RV (Ustups et al., 2013). The authors found no correlation between larval supply and recruitment in the EGB, suggesting that recruitment was regulated at the post‐settlement stage (Ustups et al., 2013). However, only a small proportion of the larvae released in the EGB would eventually be transported to the GoF, and hence, it would not be surprising if recruitment of *P. flesus *in the GoF was supply‐limited. Recruitment in flatfish is usually a reflection of density‐independent factors affecting egg and larvae at the local scale (Leggett & Frank, 1997), and shows higher variation close to the species’ distribution margin. Density‐dependent processes in the phase immediately following settlement, such as competition for space and resources in overcrowded nurseries, may also play an important role in regulating recruitment and dampening inter‐annual variability (Beverton, 1995). However, when larval supply is low, density‐dependent mortality within nurseries becomes less important and recruitment is mostly a result of density‐independent mortality during the egg and larval stages (Van der Veer, Berghahn, Miller, & Rijnsdorp, 2000). Jokinen et al. (2016) discovered that contemporary juvenile densities in the Finnish coast are much lower than those at the end of the 1970s and in the early 1980s. They hypothesized that a combination of disturbed larval supply and decreasing nursery quality due to eutrophication was the underlying cause of reduced recruitment (Jokinen et al., 2016). Our results are consistent with this hypothesis.

Marine populations are almost always open, and fluctuations in larval supply can have important effects on local population dynamics (Caley et al., 1996; Doherty, 1982; Underwood & Fairweather, 1989). Changes in environmental conditions in spawning grounds for *P. flesus* could affect larval supply to the GoF, but would not affect local populations of *P. solemdali*, and therefore provide a likely explanation for the patterns observed in this study. However, environmental conditions in the EGB are only one of the factors that are likely to shape recruitment of *P. flesus *to the GoF. Fluctuations in SSB in the EGB and changes in deep‐water currents at the time of spawning are also expected to affect, respectively, the number of larvae produced and the proportion of larvae that would reach the GoF. Unfortunately, SSB estimates for the EGB are only available from some of the years from which we have sampled cohorts (Hinrichsen et al., 2017; Orio et al., 2017; Ustups et al., 2013). Therefore, we were unable to explore the possible relationship between SSB and the decline of the proportion of *P. flesus* in the GoF. Similarly, it is possible that deep‐water currents have changed through time, affecting larval transport to the GoF, but we do not have access to data on water velocity preceding 1993. Hence, the readers should be aware of the fact that these potentially important factors may also have changed over time and be correlated with the RV in the EGB. Therefore, while the hypothesis put forward in this study is plausible and supported by the available data, further work will be needed to reach a definitive conclusion on the causes of the cessation of *P. flesus* larval subsidies to the GoF.

The timing of the shift from *P. flesus*‐dominated to *P. solemdali*‐dominated assemblages in the GoF closely mirrors the rise and fall in cod biomass in the Gulf of Riga (GoR) (Casini et al., 2012). As the GoF for the *P. flesus*, the GoR is a “true sink” habitat for cod as there are no suitable spawning grounds. Cod biomass in the GoR increased in the decade 1977–1987 as a result of a combination of larval supply from the Baltic proper as well as by active migration of juveniles and adults, but as soon as environmental conditions in the Baltic Proper became unfavorable, cod almost entirely disappeared from the GoR (Casini et al., 2012). A similar pattern was also seen in the GoF cod (e.g., Aro, 1989). Cod eggs require higher salinity than *P. flesus’ *eggs (>13 *vs.* >10 psu), yet both cod's and *P. flesus’ *RV in the EGB greatly increased following the multiple inflows of high salinity, oxygen saturated waters (MBI, Major Baltic Inflow) from the North Sea into the deep Baltic Sea basins in the 1970s (Karaseva & Zezera, 2016; Nissling et al., 2002). Hinrichsen et al. (2017; 2018) used a hydrodynamic model coupled with Lagrangian simulations to estimate the effect of fluctuating [O_2_] and salinity on flounder reproductive success and egg and larval survival in the central Baltic. The authors discovered that the EGB had the highest intra‐ and inter‐annual variability in spawning success as well as egg and larval survival, and that while most released larvae are locally retained, propagules from the EGB are also transported northward (though the GoF was not included in their simulations). Altogether, these results suggest that fluctuations in environmental conditions in the EGB may have far‐reaching effects in shaping the abundance of important fishery stocks in the GoR and the GoF.

Jokinen et al. (2015) discovered that the flounder stock on the Finnish coast has declined dramatically (75%–95% drop in abundance) since 1975. The first decline (60%) occurred in the period 1975–1997, coinciding with the temporal shift in dominance from *P. flesus* to *P. solemdali* observed in this study. The subsequent decline, which largely happened in the early 2000s, likely reflects the decline of *P. solemdali*, which reproduce locally and are better adapted to the low salinity conditions of the GoF (Nissling et al., 2002). Jokinen et al. (2016) found a negative correlation between vegetation cover in shallow nurseries and juvenile flatfish occurrence, suggesting that eutrophication has decreased the value of local nurseries. This interpretation suggests that we are not witnessing the gradual decline of a single flounder stock, but rather the successive decline of two distinct, closely related species of flounders. This scenario bears resemblance to cases of fishery collapse caused by the successive disappearance of previously unobserved, demographically independent stock components, such as the collapse of the West Greenland cod fishery (Bonanomi et al., 2015). It is not entirely clear whether the low proportion of *P. flesus* and the low commercial landings and CPUE recorded in the past three decades in the GoF are symptoms of a transient phase of unfavorable environmental conditions or are entirely a result of anthropogenic habitat degradation. Nevertheless, these results raise concern over the future of *P. flesus* and *P. solemdali *in the GoF.

## 5 CONCLUSION

What had been previously considered as a single‐stock fishery is in reality composed of two distinct species of flounders, each of which can dominate local assemblages at different points in time. The use of historical samples allowed us to discover a hidden turnover of flounder assemblages and to demonstrate this turnover coincided temporarily both with the decline in flounder stock and with the decline of environmental conditions in the northernmost spawning grounds of *P. flesus*. In the GoF, *P. flesus* has almost completely disappeared before scientists and managers even noticed their presence. The decrease in flounder abundance in the GoF does not reflect the gradual decline of a single population, but rather the disappearance of *P. flesus* and the successive decline of *P. solemdali*, which in turn are determined by the degradation of environmental conditions at local and regional scales. Our results highlight the importance of studying spatiotemporal changes in the relative contribution of different stocks to mixed‐stock fisheries, and the underlying environmental causes, to manage marine resources in the age of rapid anthropogenic change. Of particular concern is the fact that the decline of *P. flesus* in the northern Baltic Sea could have led to stronger fishing pressure on *P. solemdali*, which itself is demonstrated to have suffered from anthropogenic environmental changes (Jokinen et al., 2015, 2016). Furthermore, as the conditions for pelagic reproduction will likely continue to deteriorate as a result of ongoing environmental changes (Vuorinen et al., 2015), there are also concerns over possible local extinctions of *P. flesus* within the Baltic Sea (Momigliano, et al., 2017b). Altogether, our results call for an immediate re‐assessment of the conservation status of the two flounder species in the Baltic Sea by the International Union for the Conservation of Nature. The genetic test developed by Momigliano et al. (2018) and further refined in this study provides the means for monitoring both flounder species independently where they co‐occur, and could be employed for real‐time mixed‐stock analyses of the catch (Dahle et al., 2018). This will enable to estimate demographic changes, resilience to climate change and exploitation, and responses to management for each species separately, creating the bases for the effective adaptive management of *P. flesus* and *P. solemdali* in the Baltic Sea.

## DATA ARCHIVING STATEMENT

Data are available from the Dryad Digital Repository: https://doi.org/10.5061/dryad.606fq59.

## CONFLICT OF INTEREST

None declared.
