# Supplementary material for: Cryptic temporal changes in stock composition explain the decline of a flounder (Platichthys spp.) assemblage
Source: Evol Appl. 2019 Jan 21;12(3):549–59. doi: 10.1111/eva.12738 (PMC6383698; doi:10.1111/eva.12738)
Supplement: Supplementary file 1 [file EVA-12-549-s001.pdf]

Supplementary materials for : Cryptic temporal changes in stock composition explain the decline of a flounder (*Platichthys* spp.) assemblage

DNA EXTRACTION PROTOCOL FOR OTOLITH SAMPLES

DNA was extracted from each otolith sample using a modified salting' out protocol (Sunnucks and Hales 1996), but with highly reduced concentration of both Ethylenediaminetetraacetic acid (EDTA) and Sodium dodecyl sulfate (SDS) with respect to Sunnucks and Hales (1996) since EDTA and SDS can compromise both the physical structure and affect the chemical composition (particularly Mg) of the otoliths, hence precluding further analyses on the otolith samples following DNA extraction (Cuvelier et al, 2009; Therkildsen et al, 2010).

1- Individual otoliths, along with any attached mucous and/or dried blood were placed in 1.5 ml microcentrifuge tubes containing 580 µl of TNES buffer with low EDTA and SDS concentrations (50 mM Tris pH 8, 400 mM NaCl, 1 mM IEDTA, 0.5% SDS).

2- Otoliths was incubated at 55 C for 3 h, with gentle shaking (200 rpm) on a benchtop thermo-shaker.

3- Following incubation, 170 µl of 5 M NaCl were added and the samples were vortexed for 15 s and centrifuged at 4 °C and 16 000 g

4- The supernatant was transferred into new 1.5 ml microcentrifuge tubes and 770 µl of 100% EtOH. DNA was precipitated overnight at -20 °C. At this point, otoliths were washed five times in reverse-osmosis purified water, air-dried and stored for possible future microchemistry analyses.

5- Precipitated DNA was pelleted by centrifugation (16 000 g at 4 °C for 30 m), and the pellet was washed twice with 750 µl of EtOH.

6- DNA was eluted in 10 µl of tris buffer (10 mM tris ph 8).

REFERENCES

Cuveliers, E. L., Bolle, L. J., Volckaert, F. A. M., & Maes, G. E. (2009). Influence of DNA isolation from historical otoliths on nuclear–mitochondrial marker amplification and age determination in an overexploited fish, the common sole (Solea solea L.). Molecular Ecology Resources, 9(3), 725-732.

Sunnucks, P., & Hales, D. F. (1996). Numerous transposed sequences of mitochondrial cytochrome oxidase I-II in aphids of the genus Sitobion (Hemiptera: Aphididae). *Molecular Biology and Evolution*, *13*(3), 510-524.

Therkildsen, N. O., Nielsen, E. E., Hüssy, K., Meldrup, D., & Geffen, A. J. (2010). Does DNA extraction affect the physical and chemical composition of historical cod (*Gadus morhua*) otoliths?. ICES Journal of Marine Science, 67(6), 1251-1259.

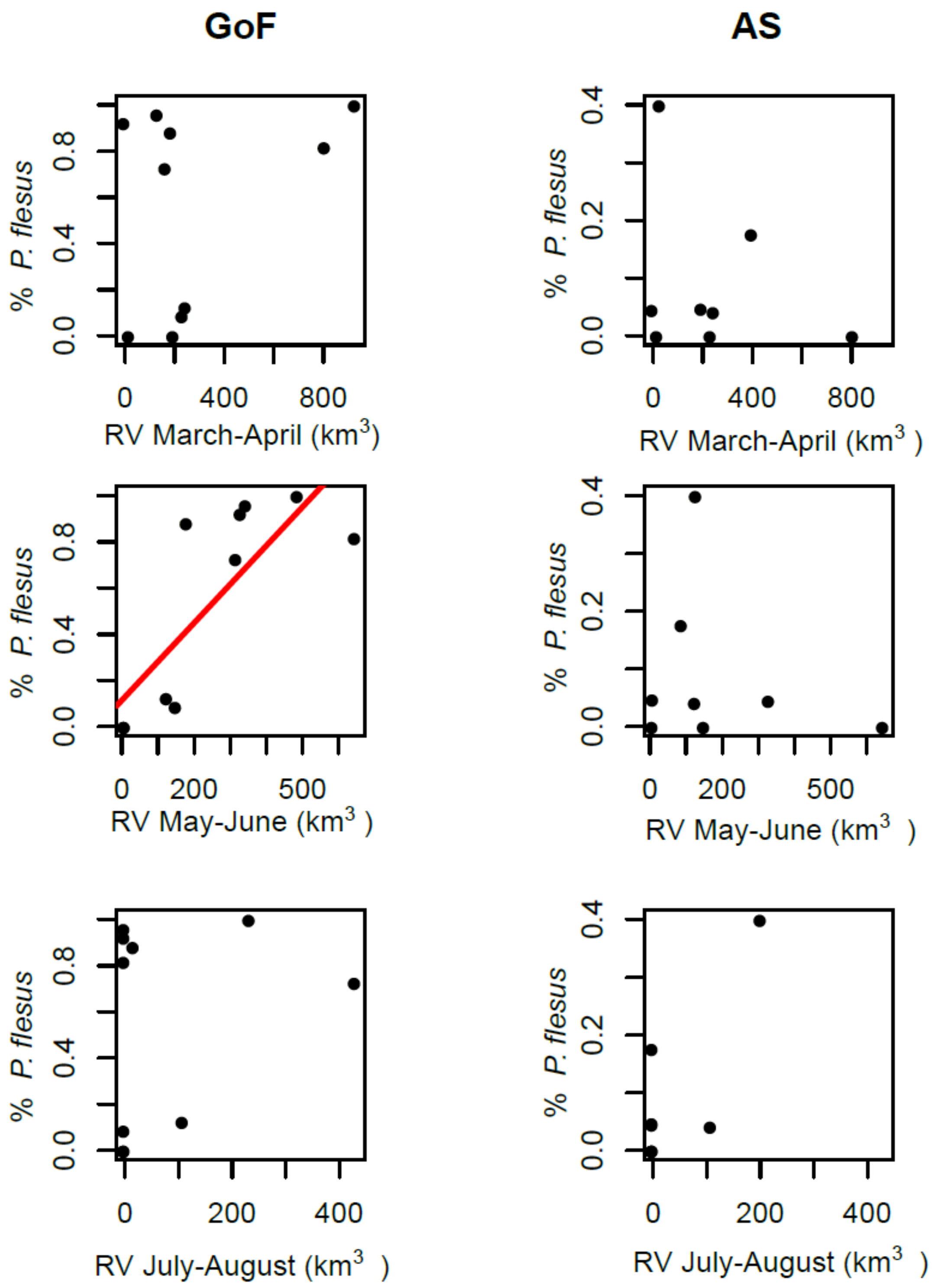

Supplementary Figure S1: Relationship between reproductive volume in the EGB (x-axis) in March-April (first row) May-June (second row) and July-August (third row) and the proportion of *P. flesus* (y-axis) in the Gulf of Finland (GoF, left) and the Åland Sea (AS, right). The only statistically significant relationship was between the EBG reproductive volume in May-June and the proportion of pelagic flounders in the GoF (center-left graph, Bonferroni adjusted p=0.042, adjusted R^2=0.57)

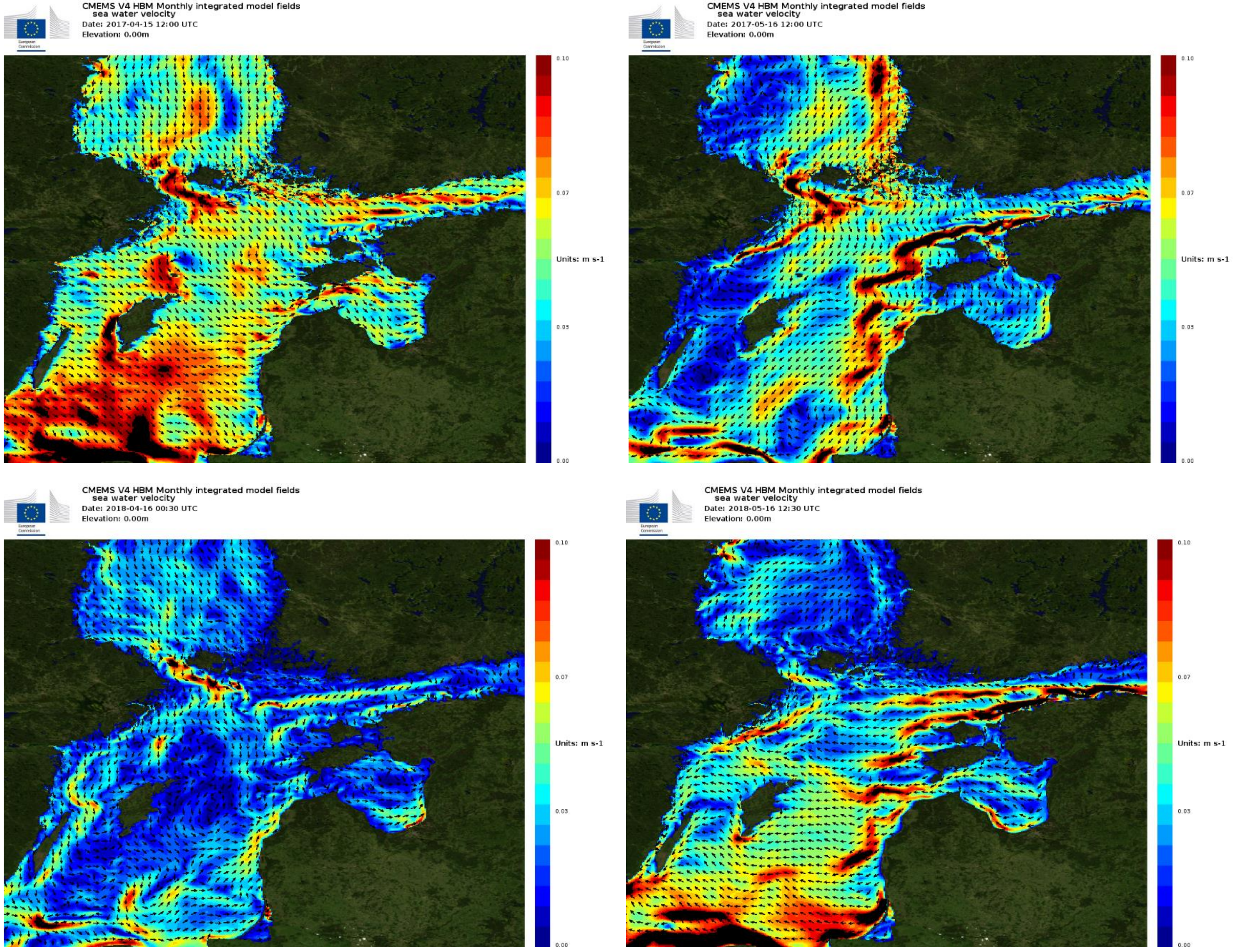

Supplementary Figure S2: Surface water velocity in spring (April and May) 2017 and 2018. Data from E.U. Copernicus Marine Service Information (http://marine.copernicus.eu/)

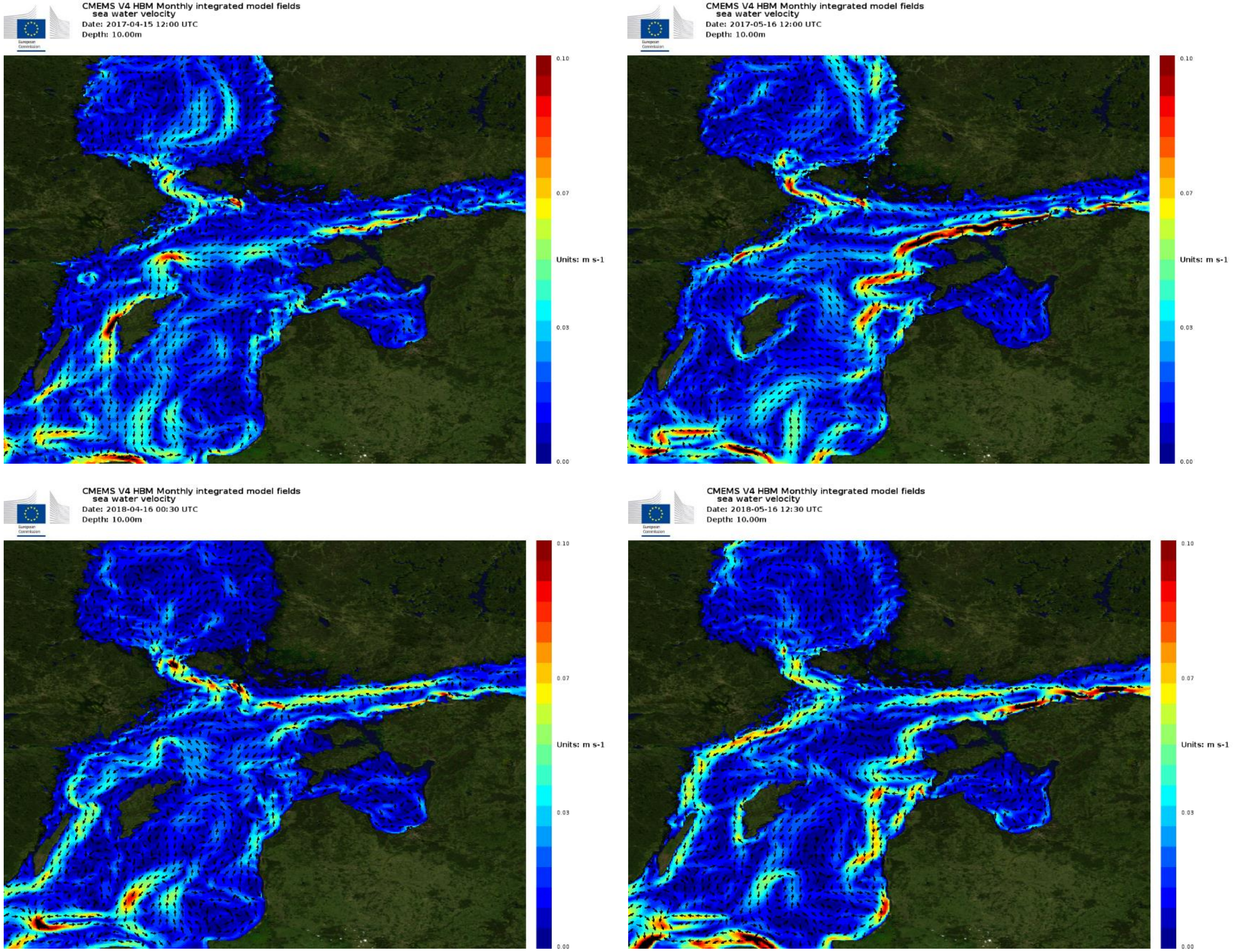

Supplementary Figure S3: Water velocity at 10m in spring (April and May) 2017 and 2018. Data from E.U. Copernicus Marine Service Information (http://marine.copernicus.eu/)

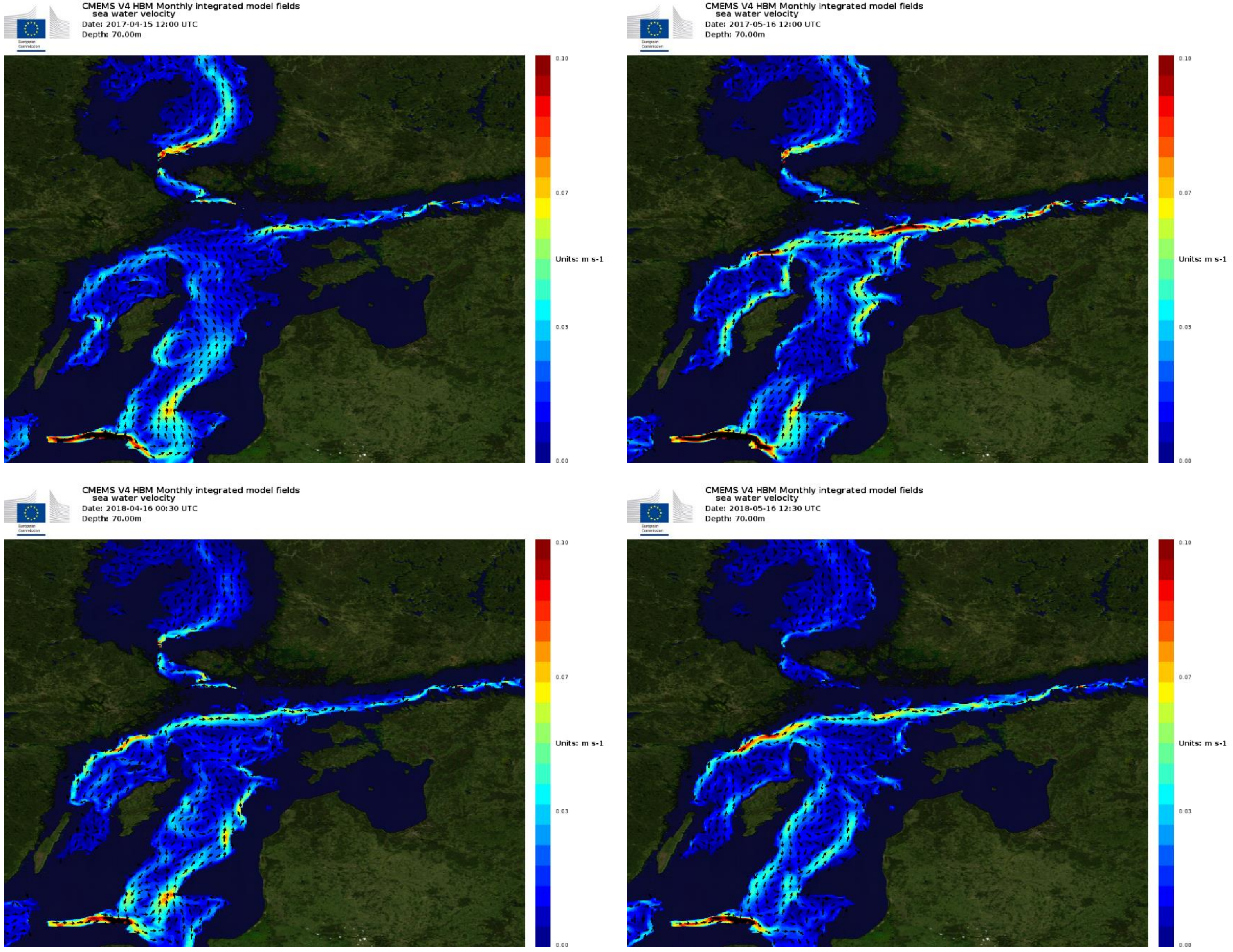

Supplementary Figure S4: Water velocity at 70 m in spring (April and May) 2017 and 2018. Data from E.U. Copernicus Marine Service Information (http://marine.copernicus.eu/)

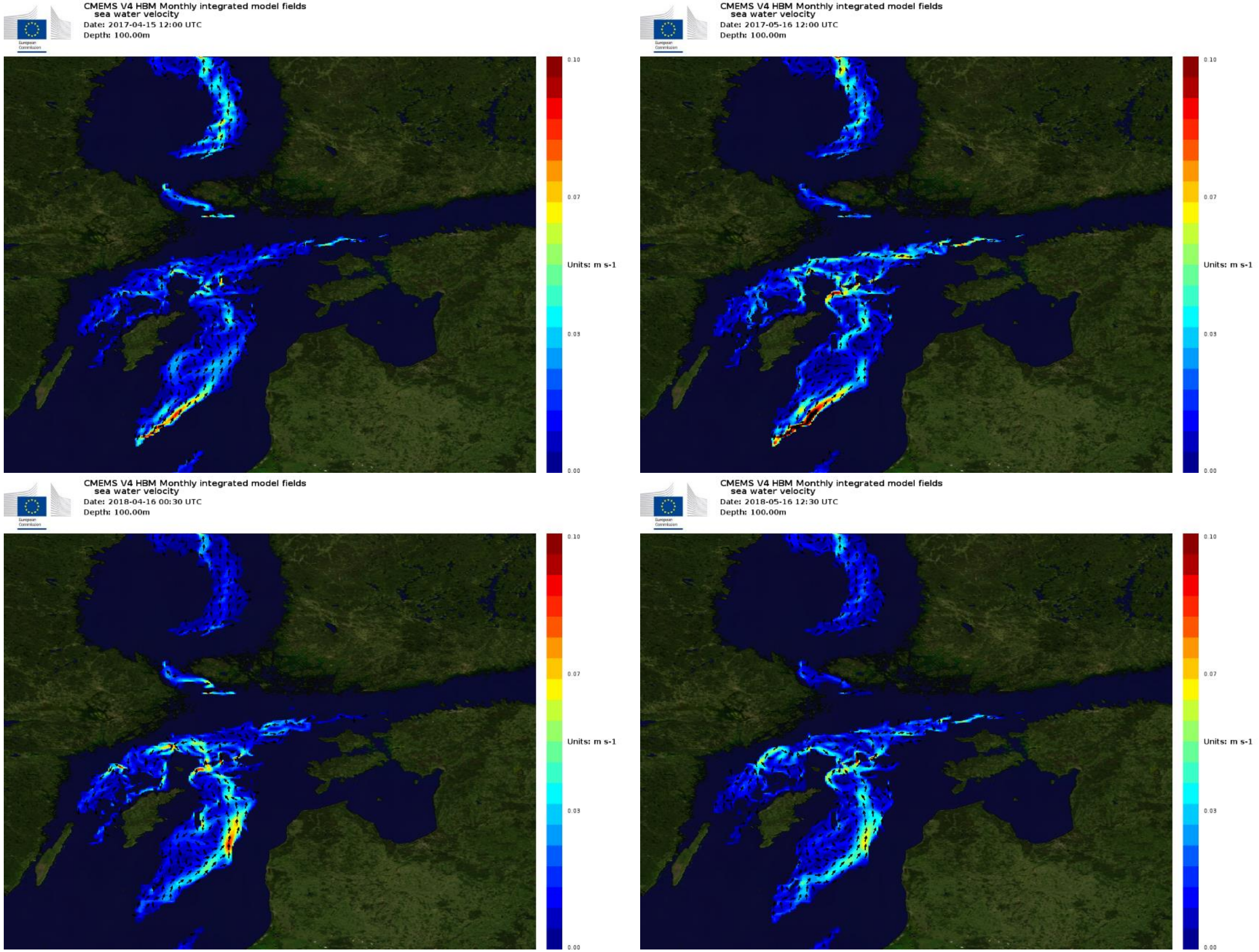

Supplementary Figure S5: Water velocity at 100 m in spring (April and May) 2017 and 2018. Data from E.U. Copernicus Marine Service Information (http://marine.copernicus.eu/)
